# Should anti-EGFR mAbs be discontinued for conversion surgery in untreated right-sided metastatic colorectal cancer? A systematic review and meta-analysis

**DOI:** 10.1186/s12957-018-1502-7

**Published:** 2018-10-08

**Authors:** Datian Chen, Xiang Zhang, Guangyi Gao, Lili Shen, Jiaqi Xie, Xiaoping Qian, Huiyu Wang

**Affiliations:** 1 Department of Oncology, Haimen People’s Hospital, Haimen, China; 2 Nanjing Drum Tower Hospital, Clinical College of Nanjing Medical University, Nanjing, China; 3 The Comprehensive Cancer Center, Nanjing Drum Tower Hospital Clinical College of Traditional Chinese and Western Medicine, Nanjing University of Chinese Medicine, Nanjing, China; 4 The Comprehensive Cancer Center of Drum Tower Hospital, Medical School of Nanjing University and Clinical Cancer Institute of Nanjing University, Nanjing, China; 5 Wuxi People’s Hospital affiliated to Nanjing Medical University, Wuxi, China

**Keywords:** Metastatic colorectal cancer, Primary tumor location, Anti-EGFR mAb, Resection, Conversion therapy

## Abstract

**Background:**

Previous studies have demonstrated that left-sided tumors have better prognoses than right-sided tumors in RAS wild-type mCRC (metastatic colorectal cancer) patients, while anti-EGFR mAbs appear to have no advantage compared with bevacizumab for right-sided tumors in these patients. Nevertheless, it remains unclear whether primary tumor location affects patients’ options for potentially curative resection.

**Methods:**

PubMed, the Cochrane Library, Embase, ASCO, and ESMO conference abstracts were searched. The inclusion criteria were RCT (randomized controlled trials) studies that evaluated the efficacy of anti-EGFR mAbs based on primary tumor location. The outcomes included ORR, ETS, and DpR. ORs for ORR were calculated with 95% confidence intervals by Comprehensive Meta-Analysis, version 2.0.

**Result:**

Nine studies including nine RCTs were analyzed. Regardless of left- or right-sided tumors, the ORRs for anti-EGFR mAb (left-sided: 80.2%, 95% CI, 47–95%; *I*^2^ = 76.9%; right-sided: 46.1%, 95% CI, 39.4–53.0%; *I*^2^ = 18.9%) were both higher than the control arm including chemotherapy with or without bevacizumab. The ORs for anti-EGFR mAbs have a significant benefit compared with chemotherapy with or without bevacizumab in left-sided tumors (OR = 2.19, 95% CI, 1.41–3.38; *P* < 0.001). For right-sided tumors, anti-EGFR mAbs still significantly improved the ORR compared with chemotherapy alone (OR = 1.75, 95% CI, 1.05–2.90; *P* = 0.03), and the OR numerically favored the anti-EGFR mAbs compared with bevacizumab (OR = 1.281, 95% CI, 0.77–2.12; *P* = 0.335). The data of ETS and DpR from three RCTs also favored the EGFR antibody irrespective of tumor location. Resection data on differentiating tumor locations is inconclusive. For right-sided tumors, it should be noted that median PFS and OS were comparable for patients who achieved ETS in both treatment arms.

**Conclusions:**

Anti-EGFR mAbs have advantages in the tumor shrinkage regardless of left- or right-sided tumors, which is important for conversion therapy. For right-sided tumors, anti-EGFR mAbs should remain the first choice for potentially curative resection in RAS wild-type mCRC patients. ETS may represent a subgroup of patients with right-sided tumors who might benefit from the anti-EGFR mAb.

**Electronic supplementary material:**

The online version of this article (10.1186/s12957-018-1502-7) contains supplementary material, which is available to authorized users.

## Background

Colorectal cancer remains one of the most frequently diagnosed malignant neoplasms worldwide and a leading cause of cancer death [1]. Approximately 25% of patients have liver metastases at their first diagnosis, and nearly 50% of these will develop recurrent hepatic metastases during their disease [2]. Surgically resecting the metastases remains the only potentially curative strategy; however, 80~90% of patients with hepatic metastases are initially considered unresectable at diagnosis [3]. Nevertheless, due to the availability of neoadjuvant systemic chemotherapy and advanced revolutionary surgical techniques, more patients initially considered unresectable become eligible for conversion surgery following treatment. The comparable outcomes of secondary resection to those of primary resection make resectability a preferential therapeutic goal. Adam demonstrated that neoadjuvant chemotherapy allowed 12.5% of patients with previously unresectable colorectal liver metastases be rescued by surgical resections, 38% of whom had extrahepatic metastases. In certain conditions, even patients with extrahepatic metastases can be potential candidates for secondary resection [4].

Adding biologic agents to chemotherapy may further improve conversion therapy rates in patients with RAS wild-type mCRC. At present, the data seems to support a high-priority use of anti-EGFR mAb when combined with standard doublet chemotherapy regimens (FOLFIRI or FOLFOX) for converting previously unresectable metastases to potentially curative resection [5].

Recently, increasing evidence has shown that tumors arising from different sides of the colon have diverse molecular and clinical characteristics [6, 7]. Right-sided tumors are more commonly related to RAS and BRAF mutations and are CIMP-high with microsatellite instability [8–10]. Conversely, left-sided tumors are more often associated with HER2 amplification, chromosomal instability, and gene expression profiles that improve anti-EGFR mAb outcomes in patients with RAS wild-type mCRC [6, 8, 10, 11]. Two meta-analyses have shown that chemotherapy plus EGFR antibody have superior treatment outcomes compared with chemotherapy with or without bevacizumab in RAS wild-type left-sided tumors, while adding bevacizumab was numerically associated with better survival in right-sided tumors [12, 13]. Primary tumor location may help decide the treatment since it is prognostic and predicts therapeutic response. However, whether the tumor location affects the choice of targeted drugs for conversion therapy remains unclear. Because the results from 2 phase II trials showed no significant differences were observed in early tumor shrinkage and objective response rate between the tumor sidedness  when cetuximab was combined with chemotherapy [14, 15]. The addition of cetuximab significantly increased the secondary resectable rate compared with chemotherapy alone [16, 17]. Thus, the impact of primary tumor sidedness on resection rates for previously unresectable metastatic CRC must be determined as well as how this connects to using targeted agents. However, resectability data are limited.

Notably, tumor response rate and resection rate have been directly correlated in studies investigating patients with unresectable colorectal liver metastases, mainly by the superior tumor lesion shrinkage [18, 19]. Tumor assessments beyond RECIST, including ETS (early tumor shrinkage) and DpR (depth of response), also suggest improved resectability. We therefore performed a systematic review and meta-analysis of randomized trials to calculate whether primary tumor location affects the choice of biologic agents for RAS wild-type mCRC patients with the opportunity for potentially curative resection.

## Methods

### Search strategy and selection criteria

We systematically reviewed the available data from randomized controlled trials that compared the anti-EGFR mAb with bevacizumab or chemotherapy alone based on tumor shrinkage. PubMed, Embase, and the Cochrane Library databases were searched using the following terms: colorectal, ETS, ORR, DpR, cetuximab, panitumumab, tumor sidedness, tumor location, and right-sided, left-sided, and resection. Furthermore, meeting abstracts including ASCO and ESMO were searched manually. The latest search was conducted in August 2018. To recognize additional relevant studies, all references were checked within original reports and review papers during the systematic review. Only RCT trials that reported the results evaluating EGFR antibody efficacy by tumor location in patients with RAS wild-type unresectable mCRC were retrieved. Non-English language articles were excluded. After the initial selection process, two reviewers independently screened the remaining abstracts and titles. Finally, full-text reviews were performed on studies that appeared to meet the inclusion criteria. This study followed the guidelines set by the Preferred Reporting Items for Systematic Reviews and Meta-Analyses (PRISMA) statement.

### Data extraction

Data were extracted from patients with RAS wild-type mCRC. Two reviewers reviewed all eligible publications and extracted the available data. For each study, data were obtained on the number of patients based on tumor location and study treatment per arm, ORR, ETS, DpR, and resection rate for patients with left-sided or right-sided tumors. Both assessments were performed in duplicate and a consensus was reached on all items. Data duplication was avoided by referencing the research center name and the author’s name. Investigators validated these data before the analyses.

### Statistical analysis

The primary endpoint of interest was ORR in RAS wild-type patients by treatment based on whether the primary tumor was left or right-sided. ETS and DpR were considered secondary endpoints. DpR data were unsuitable for meta-analysis due to their nature; therefore, DpR results are presented by the study. We calculated the weighted pooled ORR rates based on the clinically evaluable patients, using a random effects model to account for heterogeneity. The predictive value based on tumor location was investigated by comparing the ORs (chemotherapy plus anti-EGFR mAb) versus the control arms that were either chemotherapy plus bevacizumab or chemotherapy alone. An OR of > 1 indicated a favorable experimental arm containing the EGFR antibody. Subgroup analyses were performed based on the control arm. Statistical significance was considered when the *P* value was < 0.05. All considered tests were two-sided. Heterogeneity was evaluated by the Cochran’s *Q* test and the *I*^2^ statistic. A *P* value < 0.10 or as an *I*^2^ > 50% was defined as significant heterogeneity. We evaluated publication bias using a funnel plot analysis with Begg’s and Egger’s tests. Analyses were performed using Comprehensive Meta-Analysis, version 2.exe software (BioStat, Inc.).

## Results

The initial search included 822 articles (Additional file 1: Figure S1). By excluding duplicates and screening titles/abstracts, 9 articles, including 9 first-line RCTs, were investigated in the overall analysis. Table 1 summarizes the characteristics of the 8 studies, including 555 left-sided and 1827 right-sided RAS wild-type patients. Primary tumors originating from the rectum to splenic flexure were classified as left-sided, while tumors originating from the transverse colon to caecum were considered right-sided. It is worth noting that the CALGB 80405 omitted the transverse colon from the analysis. Four RCTs [20–23] evaluated the efficacy of anti-EGFR mAb plus chemotherapy versus chemotherapy alone by tumor location, including a study in the context of a chemotherapy triplet of FOLFOXIRI. Three RCTs [13, 20, 24] evaluated the anti-EGFR mAb plus chemotherapy versus bevacizumab plus chemotherapy. Two RCTs [25] investigated the EGFR antibody plus FOLFOX or FOLFIRI. All patients with CRC metastases were considered unresectable at the time of the study entry. Three studies [24, 26] reported the ETS and DpR by treatment based on the tumors arising from different sides. We present the clinical outcome data efficacy below with pooled analysis results.

**Table 1:** Treatment effects within subgroups defined by primary tumor location in patients with RAS wild-type metastatic colorectal cancer

Study	Intervention	Number of response	Number of patients	ORR (%)	OR	95% CI	*P* value
Right-sided colorectal cancer							
PRIME [20]	FOLFOX + panitumumab FOLFOX	16 16	38 46	42.1 34.8	1.364	0.56–3.30	0.492
TAILOR [21]	FOLFOX + cetuximab FOLFOX	20 9	45 38	44.4 23.7	2.578	0.99–6.67	0.051
CRYSTAL [22]	FOLFIRI + cetuximab FOLFIRI	1417	3351	42.4 33.3	1.474	0.60–3.64	0.4
MACRO-2/PLANET [25]	FOLFIRI/FOLFOX + panitumumab	11	33	33.3	–	–	–
VOLFI [23]	FOLFOXIRI + panitumumab FOLFOXIRI	6 3	10 8	60 37.5	2.500	0.37–16.88	0.347
PEAK [20]	FOLFOX + panitumumab FOLFOX + bevacizumab	14 7	22 14	63.6 50.0	1.75	0.45–6.83	0.42
FIRE-3 [24]	FOLFIRI + cetuximab FOLFIRI + bevacizumab	18 19	30 38	60.0 50.0	1.500	0.57–3.95	0.412
CALGB80405 [13]	FOLFIRI/FOLFOX + cetuximab FOLFIRI/FOLFOX + bevacizumb	30 31	71 78	42.3 39.7	1.109	0.58–2.13	0.756
Left-sided colorectal cancer							
PRIME [20]	FOLFOX + panitumumab FOLFOX	114 82	168 156	67.9 52.6	1.905	1.21–2.99	0.005
TAILOR [21]	FOLFOX + cetuximab FOLFOX	97 70	146 162	66.4 43.2	2.602	1.63–4.13	< 0.001
CRYSTAL [22]	FOLFIRI + cetuximab FOLFIRI	103 56	142 138	72.5 40.6	3.867	2.34–6.38	< 0.001
MACRO-2/PLANET [25]	FOLFIRI/FOLFOX + panitumumab	78	148	52.7	–	–	–
VOLFI [23]	FOLFOXIRI + panitumumab FOLFOXIRI	48 17	53 25	90.6 68	4.518	1.30–15.72	0.018
PEAK [20]	FOLFOX + panitumumab FOLFOX + bevacizumab	34 31	53 54	64.1 57.4	1.328	0.61–2.89	0.476
FIRE-3 [24]	FOLFIRI + cetuximab FOLFIRI + bevacizumab	93 78	124 133	75.0 58.6	2.115	1.24–3.60	0.006
CALGB80405 [13]	FOLFIRI/FOLFOX + cetuximab FOLFIRI/FOLFOX + bevacizumb	120 88	173 152	69.4 57.9	1.647	1.04–2.60	0.032

### Overall response rate and odds ratio for ORR

**Fig. 1:**
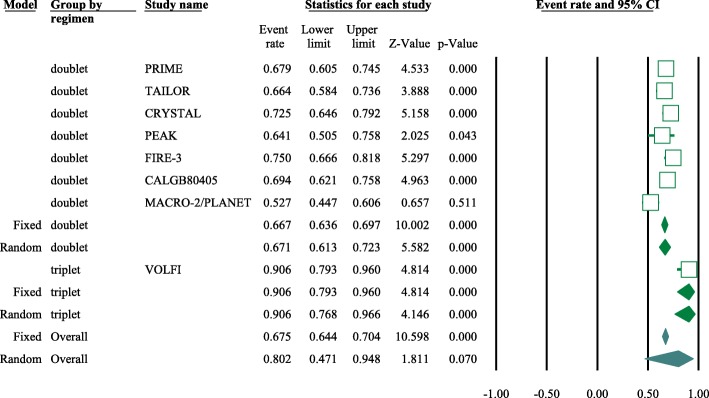
Forest plots for pooled ORR of left-sided tumors in the experimental arm. Doublet, doublet chemotherapy; triplet, triplet chemotherapy

**Fig. 2:**
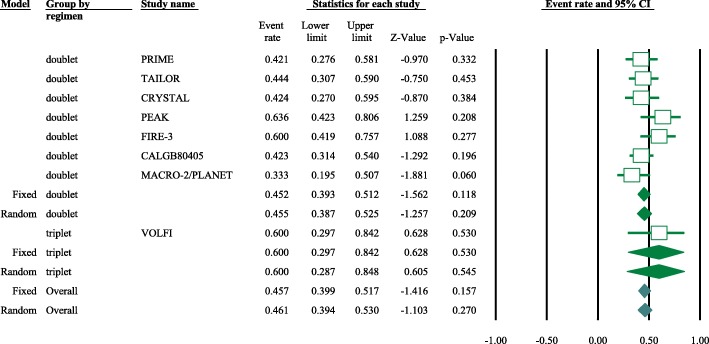
Forest plots for pooled ORR of right-sided tumors in the experimental arm. Doublet, doublet chemotherapy; triplet, triplet chemotherapy

**Fig. 3:**
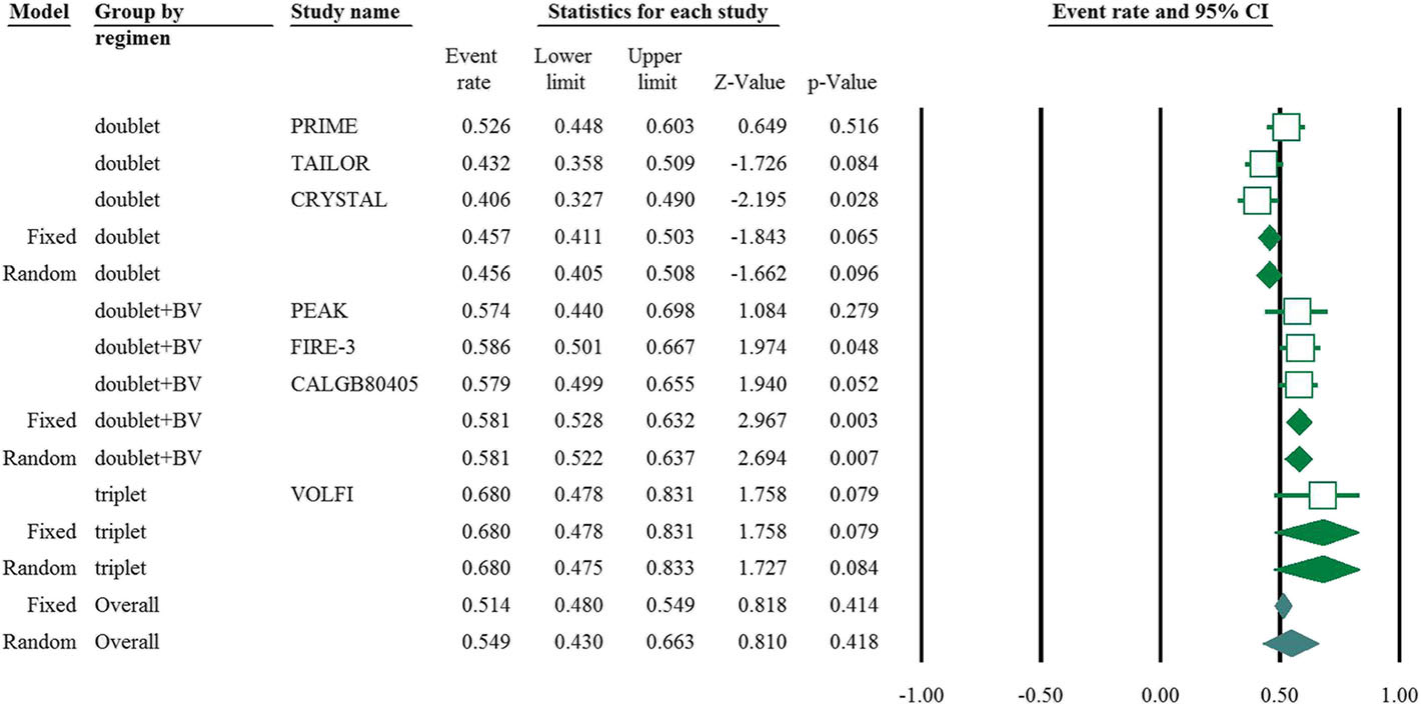
Forest plots for pooled ORR of left-sided tumors in control arm. Doublet, doublet chemotherapy; triplet, triplet chemotherapy; BV, bevacizumab

**Fig. 4:**
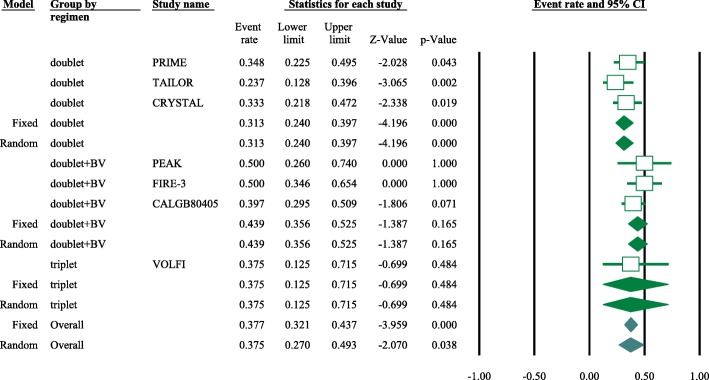
Forest plots for pooled ORR of right-sided tumors in control arm. Doublet, doublet chemotherapy; triplet, triplet chemotherapy; BV, bevacizumab

**Fig. 5:**
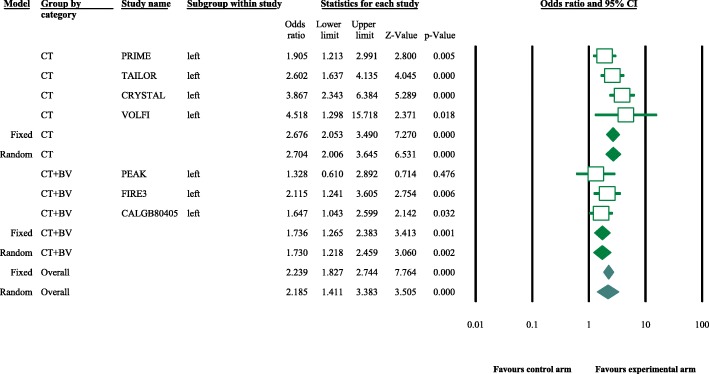
Forest plots showing odds ratio (OR) for overall response rate (ORR) comparing anti-EGFR antibody plus chemotherapy with control arm for the left-sided mCRC. CT, chemotherapy; BV, bevacizumab

**Fig. 6:**
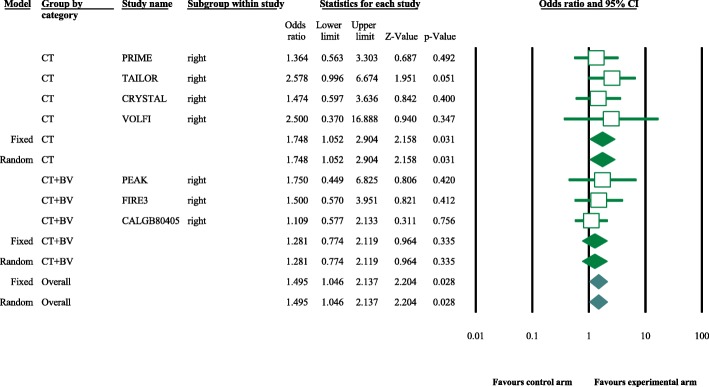
Forest plots showing odds ratio (OR) for overall response rate (ORR) comparing anti-EGFR antibody plus chemotherapy with control arm for the right-sided mCRC. CT, chemotherapy; BV, bevacizumab

**Fig. 7:**
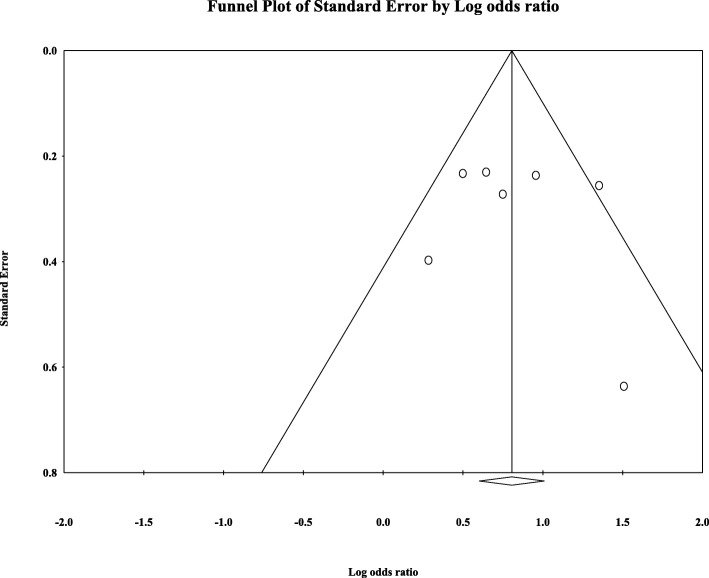
Funnel plot of publication bias for left-sided tumors

**Fig. 8:**
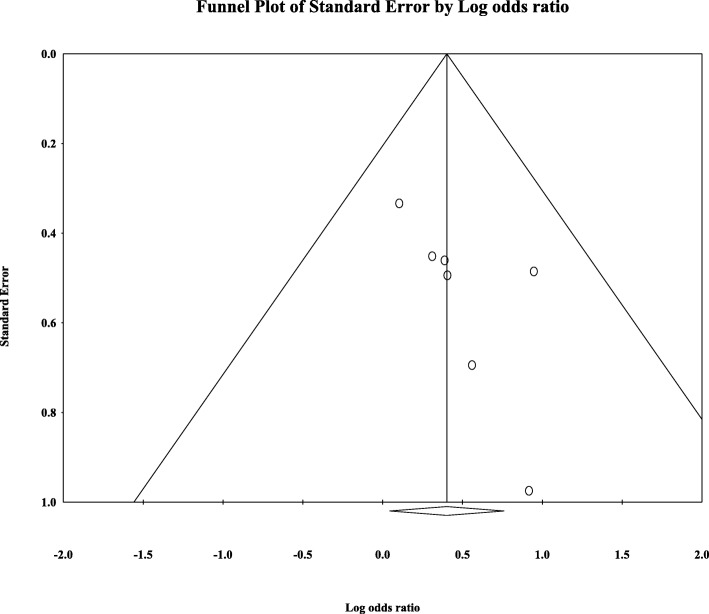
Funnel plot of publication bias for right-sided tumors

Eight articles presented ORR data (complete and partial responses) for the experimental arm (anti-EGFR mAb plus chemotherapy), with a pooled ORR of 80.2% for left-sided tumors and 46.1% for right-sided tumors (left-sided: 95% CI, 47–95%; *I*^2^ = 76.9%, Fig. 1; right-sided: 95% CI, 39.4–53.0%; *I*^2^ = 18.9%, Fig. 2, based on the random effects model). After excluding the study containing the FOLFOXIRI regimen, the pooled ORR was 67.1% (95% CI, 61.3–72.3%; *I*^2^ = 69.1%, Fig. 1) for left-sided and 45.5% (95% CI, 38.7–52.5%; *I*^2^ = 23.0%, Fig. 2) for right-sided mCRC. For the control arm, the pooled ORR was 54.9% (95% CI, 43.0–66.3%; *I*^2^ = 69.2%, Fig. 3) for left-sided tumors and 37.5% (95% CI, 27.0–49.3%; *I*^2^ = 14.9%, Fig. 4) for right-sided tumors. Subgroups were analyzed based on doublet chemotherapy, triplet chemotherapy, and doublet chemotherapy plus anti-VEGF antibody. Regardless of the side, the doublet regimen alone appeared to have the lowest ORR in the control arm. Anti-EGFR mAb plus chemotherapy greatly benefitted patients with left-sided tumors regardless of the control arm (OR = 2.19, 95% CI, 1.41–3.38; *P* < 0.001, *I*^2^ = 41.2%, Fig. 5**)**. The overall odds ratio for ORR numerically favored EGFR antibody in patients with right-sided tumors in first-line treatment of anti-EGFR versus anti-VEGF antibody combined with chemotherapy (OR = 1.281, 95% CI, 0.77–2.12; *P* = 0.335, *I*^2^ = 0%, Fig. 6). However, the benefit remained significant for right-sided tumors in anti-EGFR therapy plus chemotherapy compared with chemotherapy alone (OR = 1.75, 95% CI, 1.05–2.90; *P* = 0.03, *I*^2^ = 0%, Fig. 6). No publication bias was seen for left-sided tumors assessed by funnel plots, as per Begg’s test (*P* = 0.548) and Egger’s test (*P* = 0.753). For the right-sided tumors, no obvious publication bias was observed per Egger’s test (*P* = 0.108), but Begg’s test (*P* = 0.035) was significant (Figs. 7 and 8).

### Outcomes according to the ETS and DpR

Three RCTs [24, 26]evaluated the ETS and DpR of the anti-EGFR mAb in the first-line treatment of RAS wild-type mCRC compared with comparator treatment by tumor locations. (Table 2) In FIRE-3 and PEAK, chemotherapy plus anti EGFR mAb had higher ETS rates regardless of mCRC side (FIRE-3 71.0% vs 50.4% for left-sided, 56.7% vs 42.1% for right-sided; PEAK 58.0% vs 41% for left-sided, 55% vs 21% for right-sided). Median DpR was also better in the chemotherapy plus anti EGFR mAb versus the chemotherapy plus bevacizumab irrespective of tumor locations (FIRE-3 42.0% vs 30.8% for left-sided; 25.8% vs 17.7% for right-sided; PEAK 70% vs 48% for left-sided, 50% vs 45% for right-sided). In PRIME, chemotherapy plus anti-EGFR mAb patients achieved higher ETS rates and median DpR in patients with left-sided tumors, while ETS and median DpR were less clear in both treatment arms in patients with right-sided tumors. Due to the limited patient numbers, resectability data are not available to evaluate the predictive role of the anti-EGFR mAb based on tumor location. However, patients with left-sided tumors experienced more resections than patients with right-sided tumors. We also notice no obvious difference was observed between treatment arms regarding median PFS and OS in patients with right-sided tumors who achieved ETS. This means not all patients with right-sided tumors are non-responders to anti-EGFR mAb. Only one study supplied the hazard ratio, Thus, a meta-analysis is unavailable.

**Table 2:** ETS, DpR and outcomes according to the primary tumor location in three RCTs

Characteristic	PRIME				FIRE-3				PEAK			
	Left-sided		Right-sided		Left-sided		Right-sided		Left-sided		Right-sided	
	Pani + FOLFOX4 (*n* = 169)	FOLFOX (*n* = 159)	Pani + FOLFOX4 (*n* = 39)	FOLFOX (*n* = 49)	Cet + FOLFIRI (*n* = 124)	Bev + FOLFIRI (*n* = 133)	Cet + FOLFIRI (*n* = 30)	Bev + FOLFIRI (*n* = 38)	Pani+mFOLFOX6 (*n* = 53)	Bev + mFOLFOX6 (*n* = 54)	Pani+mFOLFOX6 (*n* = 22)	Bev + mFOLFOX6 (*n* = 14)
ETS,*n* (%)	104 (62)	57 (36)	12 (31)	15 (31)	88 (71)	67 (50.4)	17 (56.7)	16 (42.1)	31 (58)	22 (41)	12 (55)	3 (21)
Median PFS (95% CI), months	14.8 (12.5–18.5)	11.1 (9.3–13.9)	14.9 (7.4–27.2)	7.3 (5.6–11.1)	NR	NR	7.8 (2.1–20.5)	13.4 (3.8–21.2)	16.2 (13.0–20.3)	12.9 (9.3–18.6)	10.8 (5.5–15.8)	18.4 (16.6–21.4)
							HR (95%CI) 1.718 (0.832, 3.545) *P* = 0.137					
Median OS (95% CI), months	35.0 (29.8–41.9)	31.7 (23.8–38.1)	27.2 (8.0–57.4)	23.6 (7.2–34.5)	NR	NR	27.9 (18.3–37.1)	23.2 (21.0–43.7)	55.4 (41.3–63.0)	48.5 (28.9-NE)	24.6 (10.3–48.4)	26.2 (21.0–31.3)
							HR (95%CI) 1.054 (0.453, 2.453) *P* = 0.903					
No. of ETS,*n* (%)	49 (29)	87 (55)	22 (56)	27 (55)	36 (29)	66 (49.6)	13 (43.3)	22 (57.9)	20 (38)	28 (52)	7 (32)	9 (64)
Median PFS (95% CI), months	9.4 (5.8–13.8)	6.9 (5.5–7.8)	6.5 (4.0–9.9)	6.9 (3.6–11.9)	NR	NR	2.8 (1.8–5.8)	5.2 (3.1–8.6)	11.6 (7.5–16.4)	12.4 (7.4–13.0)	5.8 (3.6–9.8)	12.6 (1.8–13.8)
							HR (95%CI)1.743 (0.841,3.609) *P* = 0.129					
Median OS (95% CI), months	19.9 (13.5–27.5)	17.2 (14.2–20.7)	10.6 (6.1–22.5)	13.1 (6.1–18.8)	NR	NR	11.7 (5.9–18.9)	15.9 (13.0–24.0)	34.2 (17.3–48.0)	27.7 (21.0–32.0)	15.3 (5.8–46.1)	23.3 (6.0–29.0)
							HR (95%CI) 1.902 (0.892, 4.056) *P* = 0.0902					
Median DpR %	59	49	37	50	42	30.8	25.8	17.7	70	48	50	45
Any resection, *n* (%)	25 (15)	21 (13)	4 (10)	6 (12)	NR	NR	NR	NR	9 (17)	10 (19)	2 (9)	1 (7)
R0 resection, *n* (%)	19 (11)	16 (10)	2 (5)	1 (2)	NR	NR	NR	NR	7 (13)	6 (11)	1 (5)	1 (7)

ETS status was unknown for some patients only FIRE-3 reported the HR for PFS and OS

*NR* not reported, *NE* not evaluable, *cet* cetuximab, *pani* panitumumab, *bev* bevacizumab

## Discussion

Distinct differences between left- and right-sided colorectal cancer led to different prognoses. However, for patients intending to undergo radical resection, surgical resection of the left and right-sided mCRC (including mCRC with liver metastases) is the only option for a potential cure and the most important factor that affects prognosis. In this systematic review and meta-analysis, we saw no difference in EGFR antibody levels between the right- or left-sided tumors, and both had higher ORRs than chemotherapy alone. Compared with bevacizumab, anti-EGFR mAbs significantly benefit the left-sided tumors, and the OR for overall response rates in right-sided tumors also show a numerical advantage from chemotherapy plus EGFR antibody compared with chemotherapy plus bevacizumab. Unfortunately, data are limited that specifically address the tumor location’s impact on conversion therapy relative to the resection rates. Thus, a definite conclusion cannot be determined. Yet, it should be noted that median PFS and OS were comparable for patients who achieved ETS in both treatment arms, which means ETS may screen a group of patients with right-sided tumors who might respond to the anti-EGFR mAb.

Although an optimal regimen for right-sided mCRC in a neoadjuvant setting is unestablished, a regimen with a high ORR should be chosen. Among these clinical trials, the Chinese BELIEF study, which evaluated the efficacy of adding cetuximab to chemotherapy (FOLFOX or FOLFIRI) as first-line treatment in patients with colorectal liver metastases compared to chemotherapy alone, may be of paramount importance. The cetuximab combination significantly increased the secondary resectable rate compared with chemotherapy alone (25.7% vs 7.4%). Patients in the cetuximab plus chemotherapy arm experienced greater objective response rates (57.1% vs 29.4%; *P* < .01) and longer survival (median 30.9 vs 21.0 months). Upon assessing the extended RAS mutation status, the cetuximab-induced therapeutic effects were numerically more pronounced [16, 17]. Another clinical trial, CELIM, confirmed the value of conversion chemotherapy managed within a multidisciplinary team and demonstrated a superior outcome in patients with unresectable liver-limited metastases involving a neoadjuvant treatment followed by liver metastasis resection. Both regimens (cetuximab plus FOLFOX or FOLFIRI) yielded high responses and increased resection rates [27, 28]. The PLANET study, which evaluated panitumumab plus FOLFOX or FOLFIRI, had the similar results [29]. There are also studies exploring the bevacizumab in unresectable lesions that are potentially convertible to resectability. The addition of bevacizumab to irinotecan-based regimens improved the response rate, while bevacizumab showed no benefit to the oxaliplatin-based regimens with regard to the response rate [30–32]. Despite the lack of direct data comparing first-line anti-EGFR mAb with bevacizumab in the conversion setting for potentially curative resection in RAS wild-type mCRC, higher ORRs were observed in right-sided patients treated with anti-EGFR mAb. This promotes the idea that in RAS wild-type patients with right-sided tumors, EGFR antibody should remain the first choice for conversion therapy.

Superior novel response-related endpoints are being investigated in mCRC trials to measure temporal and quantitative tumor burden alterations beyond those provided by RECIST. Early tumor shrinkage (ETS), which is defined as an approximately 20% reduction in the sum of the largest tumor lesion diameters evaluated during early radiological assessment after 6–8 weeks from baseline, represent a good prognostic factor in colorectal cancer [33], while DpR assesses the maximum change in tumor size achieved during treatment [34]. In the FIRE-3 study, ETS and DpR, acquired by centralized radiological review, were both associated with improved overall survival irrespective of treatment (FOLFIRI plus cetuximab vs with FOLFIRI plus bevacizumab) in the RAS wild-type population [35]. Furthermore, FOLFIRI plus cetuximab enhanced ETS and DpR compared with the FOLFIRI plus bevacizumab group. Retrospective analyses from the CRYSTAL and OPUS clinical trials also revealed that the cetuximab combinations to first-line chemotherapy enhanced the ETS and DpR frequencies, and these parameters were linked with long-term outcomes in mCRC patients [36, 37].

Clinically obtaining the ETS and maximal DpR will likely exclusively benefit patients who are potential candidates for conversion resection. In the PRIME and PEAK studies, patients receiving panitumumab had higher ETS rates and greater DpR than those without panitumumab [38]. ETS and DpR improved PFS, OS and resection rates. Most resections occurred in patients from the highest DpR categories. The randomized phase 2 trial, PLANET, reported a head-to-head trial of panitumumab plus FOLFOX4 versus panitumumab plus FOLFIRI in the first-line treatment of mCRC and showed both regimens have a high ETS and ORR, allowing potentially curative resection [38]. Shrinkage should be achieved early to allow resection in potentially resectable patients as soon as possible to avoid surgical complications from prolonged treatment or potential liver toxicities.

Although the ORR, ETS and DpR favored the EGFR antibody in right-sided tumors for RAS wild-type mCRC patients, left-sided tumors had better prognoses regardless of treatment. The molecular differences in BRAF and NRAS mutations and CIMP-high and gene expression in tumor sidedness may account for this since the effect of primary tumor location was not significant on multivariate analysis [39]. Recently, the four consensus molecular subtypes (CMSs) emerged with marked differences: CMS1 (MSI immune), CMS2 (canonical), CMS3 (metabolic) and CMS4 (mesenchymal). The important biologic distinctions may explain the differential responses to targeted therapy between primary tumor locations [40, 41].

Luckily, triplet chemotherapy can overcome this obstacle. In the TRIBE study, FOLFOXIRI plus bevacizumab improved mCRC patient outcomes compared with the FOLFIRI plus bevacizumab, and the treatment effect was unaffected by BRAF and RAS status [42]. Interestingly, a pooled analysis assessing the efficacy of FOLFOXIRI plus bevacizumab demonstrated a 69% ORR and 39.1% surgical conversion [43]. In our study, adding panitumumab to FOLFOXIRI increased the overall response rate from 60.0 to 90.6% in left-sided tumors and 37.5 to 60% for right-sided tumors in patients with RAS wild-type unresectable mCRC. The overall resections were 60% versus 36.4% (FOLFOXIRI plus panitumumab vs FOLFOXIRI). Thus, triplet chemotherapy may be the preferred therapy option for right-sided tumors, but considering the toxicity, the standard doublet chemotherapy is more rational.

We acknowledge several limitations to these analyses. First, most data were derived from retrospectively analyzing radiologic imaging rather than a formal, prospective, analysis at fixed, pre-defined time points. Many of the presented data were derived from abstract-only presentations, even if they referred to large, historical, randomized trials with long follow-ups. Second, the patients analyzed are only an unselected metastatic population; thus, the ORR may have differed in metastasis. Furthermore, as we could not access patient-level data from all studies, only a study-level meta-analysis of ORR could be performed, and only three studies evaluated the ETS and DpR. Most importantly, resection data were limited for the tumor location; however, the change in tumor size related to the conversion surgery directly. Thus, we believe that good tumor response increases the resectability rate.

## Conclusion

In conclusion, our findings suggest that anti-EGFR mAb plus chemotherapy may offer better tumor shrinkage than chemotherapy alone or combined with bevacizumab in patients with RAS wild-type mCRC regardless of tumor location, which may translate into consistent probabilities for undergoing secondary resection. ETS may screen a group of patients with right-sided tumors who might respond to the anti-EGFR mAb.Therefore, more prospective RCTs are urgently needed to confirm the optimized conversion strategy for right-sided mCRC.

## Additional file

Additional file 1: **Figure S1.** Flow chart showing literature search and study. (PDF 87 kb)

## Abbreviations

DpR: Depth of response
ETS: Early tumor shrinkage
ORR: Objective response rate
