# Supplementary figures and images for: Should anti-EGFR mAbs be discontinued for conversion surgery in untreated right-sided metastatic colorectal cancer? A systematic review and meta-analysis
Source: World J Surg Oncol. 2018 Oct 8;16:200. doi: 10.1186/s12957-018-1502-7 (PMC6176519; doi:10.1186/s12957-018-1502-7)

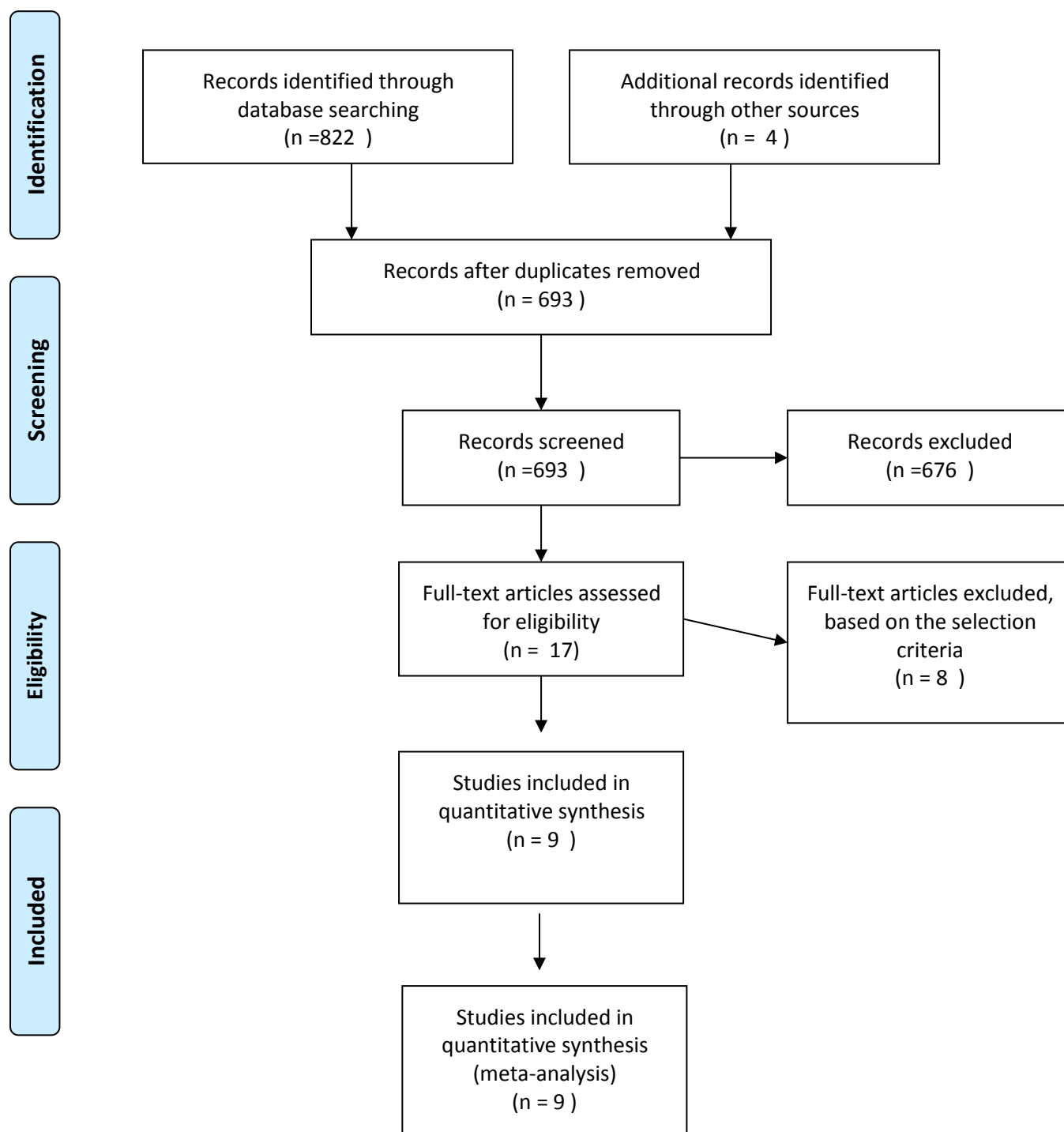

**Figure S1.** Flow chart showing literature search and study selection.

Supplement: Supplementary file 1 — Figure S1. Flow chart showing literature search and study. (PDF 87 kb) [file 12957_2018_1502_MOESM1_ESM.pdf]
